# Combing High-Modulus Fibers with a Novel Foaming Structure Applied to Protective Sandwich-Structured Composites: Manufacturing Techniques and Property Evaluations

**DOI:** 10.3390/polym15020424

**Published:** 2023-01-13

**Authors:** Yi-Huan Ho, Yan-Yu Lin, Mei-Chen Lin, Ching-Wen Lou, Yueh-Sheng Chen, Jia-Horng Lin

**Affiliations:** 1 Laboratory of Fiber Application and Manufacturing, Advanced Medical Care and Protection Technology Research Center, Department of Fiber and Composite Materials, Feng Chia University, Taichung 407102, Taiwan; 2 Department of Biomedical Engineering, College of Biomedical Engineering, China Medical University, Taichung 404333, Taiwan; 3 Fujian Key Laboratory of Novel Functional Fibers and Materials, Minjiang University, Fuzhou 350108, China; 4 Department of Bioinformatics and Medical Engineering, Asia University, Taichung City 413305, Taiwan; 5 Department of Medical Research, China Medical University Hospital, China Medical University, Taichung 404327, Taiwan; 6 Innovation Platform of Intelligent and Energy-Saving Textiles, School of Textile Science and Engineering, Tiangong University, Tianjin 300387, China; 7 Advanced Medical Care and Protection Technology Research Center, College of Textile and Clothing, Qingdao University, Qingdao 266071, China; 8 School of Chinese Medicine, China Medical University, Taichung 404333, Taiwan; 9 College of Material and Chemical Engineering, Minjiang University, Fuzhou 350108, China

**Keywords:** sandwich structure, polyurethane (PU) foam, Kevlar woven fabric, carbon fibers, electromagnetic wave shielding effectiveness (EMSE)

## Abstract

This study proposes the composites with a sandwich structure that is primarily made by the multi-step foaming process. The staple material is polyurethane (PU) foam that is combined with carbon fibers, followed by a Kevlar woven fabric. The composites are evaluated in terms of puncture resistance, buffer absorption, and electromagnetic wave shielding effectiveness (EMSE). The manufacturing process provides the composites with a stabilized structure efficiently. Serving the interlayer, a Kevlar woven fabric are sealed between a top and a bottom layer consisting of both PU foam and an aluminum film in order, thereby forming five-layered composites. Namely, the upper and lower surfaces of the five-layered sandwiches are aluminum films which is laminated on a purpose for the EMSE reinforcement. The test results indicate that the PU foam composites are well bonded and thus acquire multiple functions from the constituent materials, including buffer absorption, puncture resistance, and EMSE. There is much prospect that the PU foam composites can be used as a protective material in diverse fields owing to a flexible range of functions.

## 1. Introduction

With the progress of technology, to lessen the product weight is a global pursuit prior to other essential elements. Composites that are reinforced by fibers could attain high strength, high rigidness, ease of design, a light weight [1], and a higher maximum load, so they have been commonly used to build the main structures of aircrafts. Furthermore, fiber-strengthened composites are also pervasively used in other fields including automobile and motorbike, sports, energy industry, and other emerging industries, which suggests an immensely commercial potential of fiber-strengthened composites. Moreover, composites are composed of two or more than two metallic, ceramic, or polymer materials, which provides composites with a polyphase. Multiple materials compensate for certain attributes for each other, thereby achieving a synergistic effect. Unlike products made of a solo material, composites consisting of diverse materials have versatile functions that can satisfy demands of the manufacturers and scholars [2,3,4,5,6,7,8,9,10,11].

Polyurethane (PU) is a polymer that is synthesized by the polyhydroxy compound and multiple isocyanates via addition reaction. Featuring a lightweight, a low thermal conductivity, a low hygroscopicity, good elasticity, high specific, soundproof property and thermal insulation, PU has been drawing considerable attention. In the meanwhile, the public values the quality of living, which encourages the industries to change production for lightweight and eco-friendly products. For example, PU foam materials have a sheer amplitude of applications, including the construction, domestic appliance, medicine, aerospace, and sports fields [12,13,14,15,16].

Different fillers or methods can provide PU foam composites with more functions other than mechanical properties. Sivakumar et al. proposed sandwich-structured composites with PU foam and basalt fibers, thereby strengthening the insulating sheets with an innovative structure. The test results indicated that the incorporation of silicon dioxide (SiO_2_) nanoparticles improved the bending property of the composite sandwiches by 3%, compared to the control group (pure PU foam). The composite sandwiches were also mechanically reinforced with a lighter weight [17]. Chen et al. studied how the content of particles that were made of bamboo leaves was associated with the acoustic performance of PU foam. With 6% of bamboo leaf particles, the PU foam composites demonstrated the maximal noise reduction coefficient and the maximal sound absorption coefficient. Specifically, the optimal sound absorption of PU foam composites occurred when 8% of particles (2–3 mm) were incorporated [18]. Jathin et al. explored the influence of aluminum oxides over the mechanical properties of rigid PU foam. A combination of the fillers and MDI was mixed with a polyol premixture. The test results indicated that the filler-containing PU foam outperformed the pure PU foam in terms of compression resistance and bending strength [19]. Mohammadi et al. embedded 0–3 wt% of RWF in PU foam for better acoustic and mechanical properties. Compared to pure PU foam, PUF/RWF composites demonstrated significantly higher acoustic and mechanical properties [20]. Zhang et al. evaluated how the presence of multiwalled carbon nanotube (MWNT) affected the properties of PU foam. The reinforcement of MWNT was dependent on its content and the blending time with polyol, which turned the close-cell state into an open-pore state of PU foam [21]. Saha et al. combined PU foam with spherical TiO_2_, flaky nano-clay, and rod-shaped CNF separately. Comparing to the reinforcement of nano-particles, the addition of CNF contributed to the maximal reinforcement, while the addition of TiO_2_ contributed the least reinforcement [22]. By contrast, Kaur et al. reinforced rigid PU foam with glass fibers. The relative measurements were conducted as related to the engineering applications. The composites showed rigidness, tensile strength, and compression resistance that were inversely proportion to the pore size. Compared to the pure PU foam, the composites exhibited better properties that had a trend of linear growth [23].

The literature proves that PU foam composites are versatile and made with a great diversity of measures. In many studies, PU foam composites were usually mechanically strengthened by the incorporation of reinforcement fillers, and likewise, the combinations of fillers and structural designs also provided more functions. In this study, a multi-foaming process is employed to produce PU foam composite sandwiches. The advantage of using the specified process is to adhere the laminations without using chemical agents. The use of PU foam gives the composites a unique structure and processing feasibility. The resulting PU foam composite sandwiches are characterized with high strength, a light weight, and high energy absorption (buffer) efficacy. In addition, the whole process is eco-friendly, pollution-free and efficient, yet contributing to a better mechanical performances and structural stability than other foaming measures. The sandwich-structured composites have carbon fiber as the reinforcement while the core and surface separately contain Kevlar woven fabric and aluminized foils, both of which as well examined the effect over the mechanical performance and EMSE of sandwiches.

## 2. Experimental

### 2.1. Materials

Kevlar woven fabrics (TORAYCA™, Taiwan) have a yarn fineness being 3000 filaments/strand, a warp density being 12.5 ends/inch, and a weft density being 12.5 picks/inch. The high-density soft PU foam agent A is polyester polyol while the hardener agent B is isocyanate (Kuang Lung Shing Corporation, Taoyuan City, Taiwan). Carbon fibers (HTS 40, TORAYCA™, Kaohsiung City, Taiwan) have a length of 6.2 mm and a diameter of 7 μm. PET aluminum films are purchased from General Horng Corporation, Taiwan.

### 2.2. Preparation Process of PU Foam Composites

**Figure 1:**
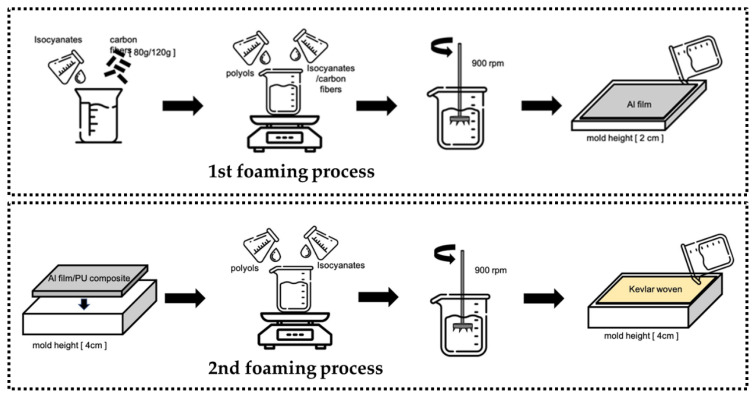
The preparation process of PU foam composites.

Figure 1 illustrates the manufacturing process of five-layered PU foam composites consisting of aluminized (Al) film/polyurethane (PU) foam/Kevlar woven fabric/PU foam/Al film planks. The planks are prepared with three stages. In the 1st stage, polyol and isocyanates with a ratio of 1:4 are blended at 900 rpm/min for 10 s, during which 80 g or 120 g of carbon fibers are added. In the 2nd stage, an aluminized film (A) is mounted in the bottom of a mold (320 mm × 330 mm × 20 mm) in advance. Next, different formulated PU (P) foaming mixtures are separately infused into the mold, followed by a Kevlar woven fabric (K). Afterwards, the mold is sealed for a 300-min foaming-curing process, forming APK composite. In the 3rd stage, the foaming mixture is formulated with polyol and isocyanates (1:4) exclusively at 900 rpm/min for 10 s. The APK composite is first placed into a mold (320 mm × 330 mm × 40 mm) with the side of Al film on the bottom. Next, the foaming mixture is infused and then covered with another piece of aluminum film. The mold is also sealed and left for the 300-min foaming and curing at room temperature. The two types of PU foam composite (experimental groups) are abbreviated as 80C and 120C according to the content of carbon fiber being 80 g and 120 g. Made with the same procedure, the control group is also five-layered PU foam composites (i.e., with a A/P/K/P/A composition) but the top PU layer does not contain carbon fibers. In the meanwhile, pure PU foam with the same thickness (40 mm) is used for comparisons and abbreviated as PU in figures. All samples along with constituent materials are tabulated in Table 1.

### 2.3. Measurements

#### 2.3.1. Drop Weight Impact Test

As specified in ASTM D 1596, the buffer absorption and the maximal absorption energy of samples is measured with a drop weight impact tester (Changfata Industrial Co., Ltd., Taichung City, Taiwan). Samples have a size of 100 mm × 100 mm. With an impact force of 9000 N, a rounded impactor is released from a specified heigh to fall free to strike the sample. Six samples from each specification are used for an average.

#### 2.3.2. Drop Weight Puncture Resistance Test

The puncture resistance of samples is measured with a drop weight impact tester (Changfata Industrial Co., Ltd., Taichung City, Taiwan) as specified in NIJ 0115.00 test standard. With an impact force of 24 J, a needle-like impactor is released from a specified height (228 mm) and falls freely to penetrate the sample. Samples have a size of 100 mm × 100 mm. Six samples from each specification are used for an average.

#### 2.3.3. Vertical Rebound Elasticity Measurement

As specified in ASTM D2632-15, a vertical rebound assembly is used to measure the rebound property of samples. The assembly is adjusted to be level for a stabilized state. The test hammer is released from the height of 400 mm in the assembly and then falls freely to strike the sample. When a sample is mounted, it is of importance to make sure that the strike site should be 14-mm from the sample rim. The first three rebounds are free from recording and only the average of the 4th, 5th, and 6th measurements is recorded and compared with the standard height of the assembly in per centage (%). The acquired ratio is called rebound rate.

#### 2.3.4. Electromagnetic Wave Shielding Effectiveness (EMSE) Measurement

As specified in ASTM D4935-10, the coaxial transmission fixture and spectrum analyzer (KC901S, TS RF Instruments Co., Ltd., Taoyuan City, Taiwan) are used to measure the EMSE of samples in a frequency range of 100 KHz~3 GHz. A blank sample with an identical thickness serves as the reference sample and its EMSE is recorded as SE_Ref_, according to which the tester is rectified. Samples have a size of 150 mm × 150 mm. Ten samples from each specification are used for an average.

## 3. Results

### 3.1. Surface Observation of PU Foam Composites

**Figure 2:**
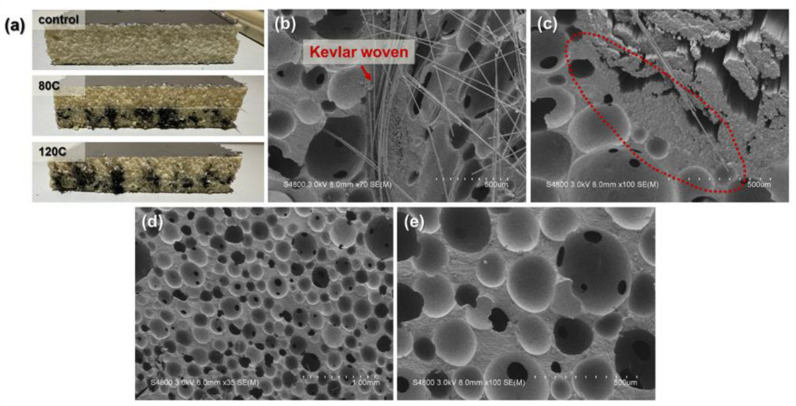
Images of PU foam composites: (**a**) cutting section; (**b**) interface among materials and (**c**) related magnified image, (**d**,**e**) SEM images of cells.

Figure 2 shows the images of PU foam composites as related to the content of carbon fibers. Figure 2a shows the 40-mm thick pure PU foam with an even foaming structure as well as the experimental groups (PU foam composites)-80C and 120C with a sandwich structure. The 80C and 120C composites have lamination layers of an aluminum film, PU foam with carbon fibers, a Kevlar woven fabric, PU foam, and an aluminum film in order. The foaming results differ for 80C and 120C groups due to the fiber content. A rise in the carbon fibers has a positive influence over the foaming density but limiting the foaming space that fibers are embedded in PU foam. The 120C group exhibits that the carbon fibers enter to another lamination layer as they are forced by the foaming pressure. Figure 2b,c show the morphology when the top aluminum films are removed from the experimental groups. The fillers and carbon fibers are well distributed and saturated in the PU foam layer, and the interfaces show that the two materials are perfectly bonded. Figure 2d,e display the PU foam as related to the magnification rate, suggesting that chemical foaming leads to the typical close-cell type porous structure with an even pore size distribution.

### 3.2. Mechanical Performances of PU Foam Composites

**Figure 3:**
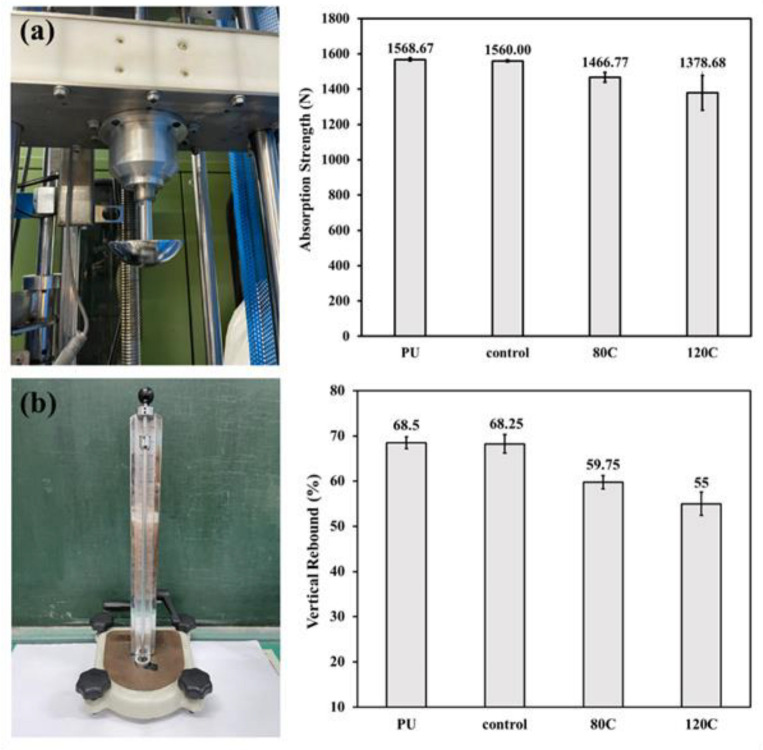
(**a**) Buffer absorption and (**b**) vertical rebound rate of PU foam composites.

In order to evaluate the buffer absorption of PU foam composites against an impact force, the drop weight impact test is conducted with a specified impact force of 9000 N. Transiently the PU foam composites bear the impact load, after which the residual forces are recorded. Figure 3a shows the impactor used in the drop weight impact test and the residual force that diverse samples react. Figure 3b shows the vertical rebound test assembly as well as the rebound rate of PU foam composites as related to the content of carbon fibers being 80 g and 120 g. When the content of carbon fibers increases from 0 g to 120 g, the cell size of PU foam is decreased and therefore the foaming density is increased, which in turn causes a decreasing trend in the rebound rate of PU foam composites [24].

**Figure 4:**
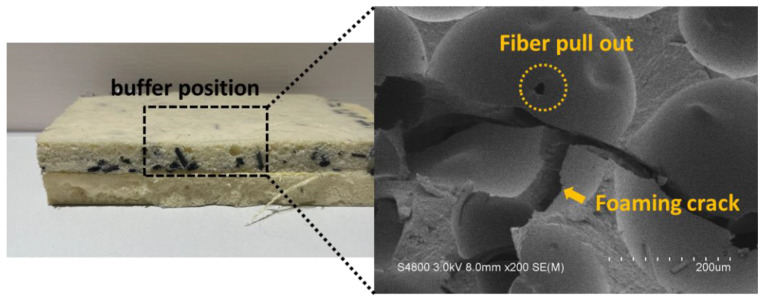
SEM images of PU foam composites after the buffer absorption test.

Figure 4 shows the damaged samples after the buffer absorption test along with the impact site where the impactor strikes. The test results indicate that the PU foam composite recovers to its primary state, which means there is little difference in the damage sites after the sample is damaged. The phenomenon is attributed to the elastic recovery of soft PU foam that demonstrates comparatively greater durability and better energy absorption than the solid materials (e.g., metal or plastic). The SEM image shows ruptured cells of the damage site. As for a multi-layered structure, PU foam layer provides the buffer efficacy and the fiber layer (reinforcement) confine the crack expansion. A small amount of fibers are dispersed in the PU foam in a non-continuous phase, and they are randomly distributed among cells. When encountering the spread of cracks, fibers stop them from exacerbation. Despite exhibiting cracks, cells are reinforced as a result of the incorporation of carbon fibers. Subsequently, the improved rigidness of whole material is beneficial to the loading support, which confines the damage rendered to the cells and cells eventually sustain the original structure.

**Figure 5:**
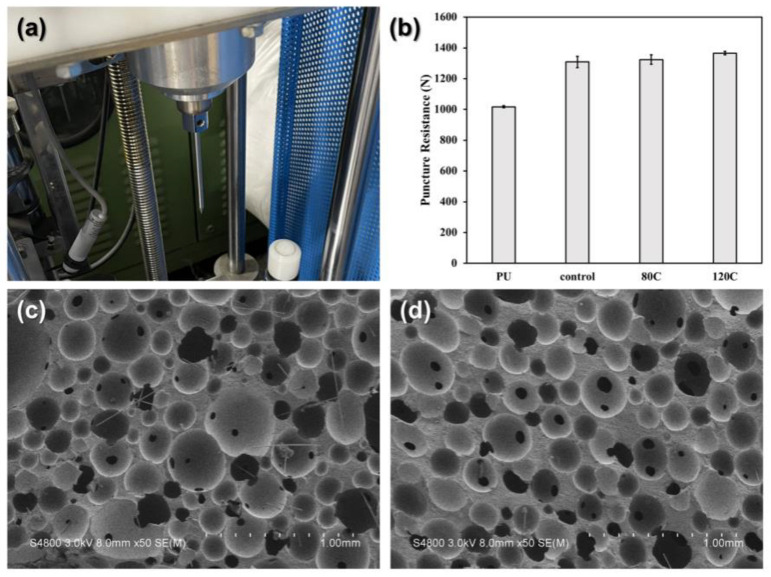
(**a**) The needle-like impactor for the dynamic penetration test and (**b**) the penetration resistance of PU foam composites. SEM images of damaged samples of (**c**) 80C and (**d**) 120C.

Figure 5 shows the penetration resistance of PU foam composites as related to the content of carbon fibers. Figure 5a shows the needle-like impactor used in the dynamic penetration. The impactor falls free to penetrate the PU foam composites, thereby examining the penetration resistance. Figure 5b exhibits the test results. A rise in the content of carbon fibers causes the penetration force to rise. Figure 5c compares the difference in the penetration resistance when 80 g and 120 g of carbon fibers are used. The foaming density is dependent on the content of carbon fibers in direct proportion.

### 3.3. Electromagnetic Wave Shielding Effectiveness (EMSE)

**Figure 6:**
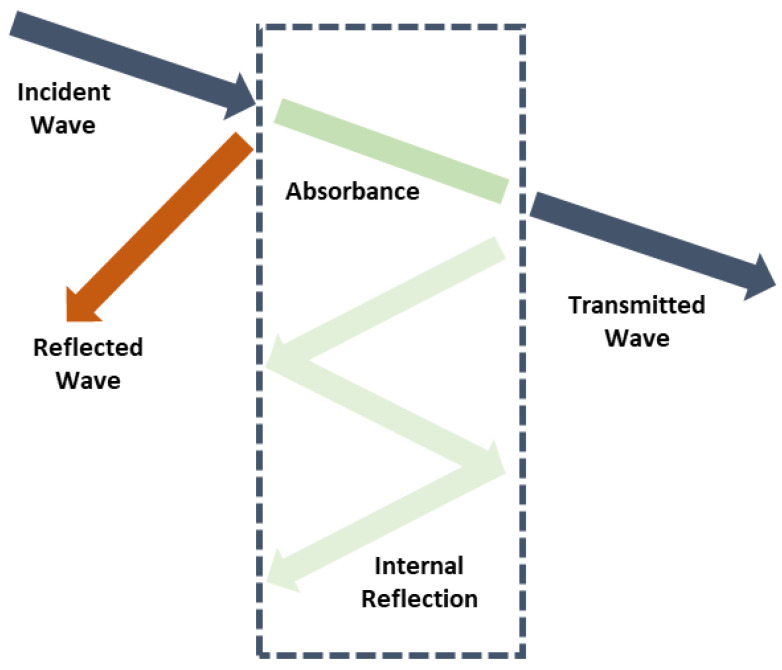
EMSE mechanisms.

**Figure 7:**
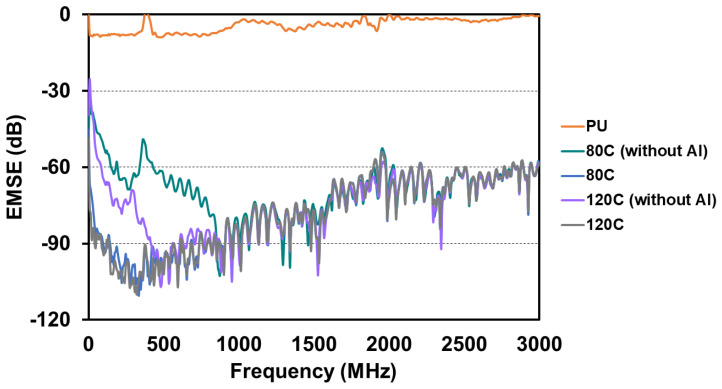
EMSE of PU foam composites.

Electromagnetic wave shielding test results are concluded to evaluate the PU foam composites. A rule of thumb, electromagnetic waves enter a shield, they are absorbed, reflected, and multiple reflected over the surface and interior of the shield before they penetrate the shield. Hence, through the different failure mechanisms as Figure 6, the energy of electromagnetic waves is consumed by the shield, which is regarded as the EMSE. In Figure 7, the top aluminum film is removed from the PU foam composites in order to evaluate the EMSE of PU foam composites as related to the content of carbon fibers. The test results suggest that the EMSE of PU foam composites changes based on the content of carbon fibers. In particular, the group containing more carbon fibers exhibits greater EMSE against electromagnetic waves at medium and low frequencies. By contrast, it also shows the EMSE of the experimental groups, including 80C and 120C, and the test results indicate that with the presence of an aluminum film, both groups exhibit considerably strengthened EMSE regardless of the content of carbon fibers. Serving as a continuous film, the aluminum film demonstrates excellent electrical conductivity, which contributes amazing EMSE that is higher than −90 dB at a frequency range of 1000~2000 MHz where other shields exhibit comparatively lower EMSE.

## 4. Discussion

Indicated by the test results, the PU foam composites made by multi-step foaming process preserve a more comparable structure stability to that of the pure PU foam. Despite comprising three diverse materials, the PU foam composites still retain an intact structure without compromised properties. Notably, because of the specific process, the size of PU foam composites allows much flexibility as required. In the PU foam, there are many evenly dispersed semi-circular cells. A rise in the content of fillers increases the viscosity of the foaming mixtures, which in turn improves the nucleation effect concurrently. Previous studies also indicated that, for the PU foam containing 24% of carbon fibers, increasing the vermiculite meant increasing the viscosity of polyol that had a positive influence over the nucleation of cells. In addition, the presence of carbon fibers reduced the energy barrier for cells, confining the occurring of nucleation over the fillers-and-polymers interface [25]. In this case, the foaming nucleation rate was on the rise. Nucleation and bubbles occurred at the same time, which subsequently reduced the gas amount demanded by the formation of bubbles [26]. By contrast, in this study, carbon fibers lead to heterogeneous nucleation while decreasing the average cell size and making the pore diameter distribution to be narrower. The more the content of carbon fibers, the smaller the pore diameter and the higher the foaming density [24].

Based on the mechanical property evaluations, the incorporation of carbon fibers is proved to mechanically strengthen the PU foam. The buffer absorption test results show that the impact load absorption rate reaches 85% regardless of the sample type. Moreover, the presence of carbon fibers has a positive influence over the buffer absorption. On one hand, when an appropriate content of carbon fibers is incorporated, carbon fibers can be embedded in PU matrices, thereby improves the interface bonding, which simultaneously increases the compression modulus and energy absorption of PU foam composites [27]. On the other hand, increasing the content of carbon fibers also enlarges the contact area between fillers and PU matrices, thereby improving the stress spreading and compression energy absorption capacity. Serving as the nucleation points for foaming reaction, carbon fibers reinforce the interaction between the fillers and PU foam as well as increase the foaming density. When a force applied externally deforms the PU foam composites, the cells are deformed, squeezed, and damaged or the cells possibly collapse. Therefore, PU foam composites with a greater foaming density have a more stabilized structure to dissipate more impact load and thus exhibit a lower residual force when being rendered with an impact force.

The PU foam composites have a decreasing trend in the rebound rate. The main factor is as follows. The foaming density is increased when the carbon fibers increase. The cells are formed due to the nucleation but the formation of cells against the mold walls is compressed and restricted from foaming because the mold reaches a density saturation. Subsequently, the PU foam has a sheath where the foaming level is lower, which leads to a higher surface density. As a result, PU foam composites exhibit a lower rebound rate when composed of more carbon fibers.

The presence of carbon fibers also contributes reinforcement in the penetration resistance of PU foam composites. PU foam composites acquire a higher density that strengthens the overall rigidity concurrently due the incorporation of carbon fibers. Regardless of the content of carbon fibers, PU foam composites demonstrate a certain level of impact resistance and greater yield stress, which in turn improves the load [22]. The results are in the conformity with the finding by Cheng et al. where they successfully strengthened the compression property of PU foam with glass fibers. Moreover, as the core of the sandwich structure, high modulus Kevlar woven fabric also plays an important role in improving the puncture resistance. When PU foam composites are penetrated by a force at a high velocity, the majority of cells collapse, and the pores become dense and at a rigid state. In the meanwhile, the Kevlar woven fabric (interlayer) and the PU foam layer are mutually squeezed to disperse the load. On the needle-like impactor penetrating the surface, the cells of the compact PU foam react with friction against the impactor, providing PU foam composites with greater puncture resistance.

The EMSE test results substantiate that carbon fibers are fully mixed with the foaming mixture and are thus evenly distributed in the PU matrices. Carbon fibers preserve electrical charges due to intrinsic electrical conductivity. With a greater content, more carbon fibers are prone to be crossed or interlaced with each other, forming a complete shielding network. The incident electromagnetic waves cause the realignment of electrons in the surface of conductive material, which attenuates the electromagnetic energy from yielding higher EMSE. Moreover, the constituent aluminum film also provides PU foam composites with good EMSE. Owing to the excellent conductivity, aluminum film of PU foam composites can reflect the majority of the incident electromagnetic waves. The incident electromagnetic waves are eventually blocked via multiple reflections by the carbon fibers from the interior of PU foam composites and core Kevlar woven fabric, thereby reinforcing the EMSE of the PU foam composites. This study proposes an innovative foaming method that bonds different materials to form PU foam composites, and successfully achieves the goal of optimization of composites.

## 5. Conclusions

Targeting at the reinforcement of PU foam, this study proposes the manufacturing process and experimental design for the versatile composites with an attainable flexible application range as well as protective functions. The five-layered sandwiches are developed based on PU foam being the matrices with the multi-step foaming process. For a pursuit of reinforcement in protective functions and flexible applications, carbon fibers (i.e., filler) are used, which improves the mechanical performances of five-layered sandwiches positively. The PU foam composites (experimental groups) are compared with the pure PU foam group and the control group in subsequent tests. According to the buffer absorption results, the experimental groups exhibit lower residual force, and the absorption strength rate exceeds 85%. As for the puncture resistance, the Kevlar interlayer of experimental groups demonstrates a positive influence, achieving a distinct protective effect. Finally, the EMSE results indicate that the PU foam composites yield amazing EMSE level that is greater than −90 dB due to the incorporation of an aluminum film as the surface layer. In conclusion, this study proposes a new prospect that combines diverse materials with an efficient multi-step foaming process, which grants the resultant products with flexible applications and a free combination of the materials corresponding to the demands. In the light of the corresponding derivative applications based on the results, the yielded EMSE and protective functions of PU foam composites can be applied to an interior material used in the hospitals and vehicles, or packaging materials for precise machines.

## Figures and Tables

**Table 1:** The specification of the PU foam composites.

Sample Types	Kevlar Woven Fabric (K)	Carbon Fiber (C)	Al Film (A)	Constituent Layers
PU	−	−	−	PU
Control	−	−	+	A/PU/K/PU/A
80C	+	80 g	+	A/PU + C (80 g)/K/PU/A
120C	+	120 g	+	A/PU + C (120 g)/K/PU/A

## Data Availability

Not applicable.

## References

[1] Carruth M.A., Allwood J.M., Moynihan M.C. (2011). The technical potential for reducing metal requirements through lightweight product design. Resour. Conserv. Recycl..

[2] Zhu C.-Y., Yu G.-L., Ren X., Huang B.-h., Gong L. (2022). Modelling of the effective thermal conductivity of composites reinforced with fibers and particles by two-step homogenization method. Compos. Sci. Technol..

[3] Shukla U., Garg K. (2023). Journey of smart material from composite to shape memory alloy (SMA), characterization and their applications—A review. Smart Mater. Med..

[4] Zhang J., Ma M., Bi Y., Liao Z., Ma Y., Huang W., Lyu P., Feng C. (2022). A review of epoxy-based composite materials: Synthesis, structure and application for electromagnetic wave absorption. J. Alloys Compd..

[5] Ibrahim I.D., Jamiru T., Sadiku E.R., Kupolati W.K., Mpofu K., Eze A.A., Uwa C.A. (2019). Production and Application of Advanced Composite Materials in Rail Cars Development: Prospect in South African Industry. Procedia Manuf..

[6] Das R., Bhattacharjee C., Altalhi T., Inamuddin (2022). Chapter 4—Green composites, the next-generation sustainable composite materials: Specific features and applications. Green Sustainable Process for Chemical and Environmental Engineering and Science.

[7] Xu X., Wang G., Yan H., Yao X. (2023). Constitutive relationship of fabric rubber composites and its application. Compos. Struct..

[8] Xu J., Ma J., Peng Y., Cao S., Zhang S., Pang H. (2022). Applications of metal nanoparticles/metal-organic frameworks composites in sensing field. Chin. Chem. Lett..

[9] Yang X., Chen Y., Zhang C., Duan G., Jiang S. (2023). Electrospun carbon nanofibers and their reinforced composites: Preparation, modification, applications, and perspectives. Compos. Part B Eng..

[10] Pham H.H., Dinh N.H., Kim S.-H., Park S.-H., Choi K.-K. (2022). Tensile behavioral characteristics of lightweight carbon textile-reinforced cementitious composites. J. Build. Eng..

[11] Wang B., Qi Z., Chen X., Sun C., Yao W., Zheng H., Liu M., Li W., Qin A., Tan H. (2022). Preparation and mechanism of lightweight wood fiber/poly(lactic acid) composites. Int. J. Biol. Macromol..

[12] Gómez-Fernández S., Günther M., Schartel B., Corcuera M.A., Eceiza A. (2018). Impact of the combined use of layered double hydroxides, lignin and phosphorous polyol on the fire behavior of flexible polyurethane foams. Ind. Crops Prod..

[13] Harith I.K. (2018). Study on polyurethane foamed concrete for use in structural applications. Case Stud. Constr. Mat..

[14] Santiago-Calvo M., Tirado-Mediavilla J., Rauhe J.C., Jensen L.R., Ruiz-Herrero J.L., Villafañe F., Rodríguez-Pérez M.Á. (2018). Evaluation of the thermal conductivity and mechanical properties of water blown polyurethane rigid foams reinforced with carbon nanofibers. Eur. Polym. J..

[15] Moon J., Kwak S.B., Lee J.Y., Kim D., Ha J.U., Oh J.S. (2019). Synthesis of polyurethane foam from ultrasonically decrosslinked automotive seat cushions. Waste Manag..

[16] Jonjaroen V., Ummartyotin S., Chittapun S. (2020). Algal cellulose as a reinforcement in rigid polyurethane foam. Algal Res..

[17] Sivakumar S., Navin Kumar B. (2022). Enhancement of bending strength of polyurethane foam reinforced with basalt fiber with silica nanoparticles in comparison with plain polyurethane foam. Mater. Today Proc..

[18] Chen S., Jiang Y. (2018). The acoustic property study of polyurethane foam with addition of bamboo leaves particles. Polym. Compos..

[19] Stanzione M., Russo V., Oliviero M., Verdolotti L., Sorrentino A., Di Serio M., Tesser R., Iannace S., Lavorgna M. (2018). Synthesis and characterization of sustainable polyurethane foams based on polyhydroxyls with different terminal groups. Polymer.

[20] Mohammadi B., Safaiyan A., Habibi P., Moradi G. (2021). Evaluation of the acoustic performance of polyurethane foams embedded with rock wool fibers at low-frequency range; design and construction. Appl. Acoust..

[21] Zhang L., Yilmaz E.D., Schjødt-Thomsen J., Rauhe J.C., Pyrz R. (2011). MWNT reinforced polyurethane foam: Processing, characterization and modelling of mechanical properties. Compos. Sci. Technol..

[22] Saha M.C., Kabir M.E., Jeelani S. (2008). Enhancement in thermal and mechanical properties of polyurethane foam infused with nanoparticles. Mater. Sci. Eng. A.

[23] Kumar M., Kaur R. (2017). Glass fiber reinforced rigid polyurethane foam: Synthesis and characterization. e-Polymers.

[24] Wang H., Li T.-T., Ren H., Peng H., Huang S.-Y., Lin Q., Lin J.-H., Lou C.-W. (2019). Expanded Vermiculite-Filled Polyurethane Foam-Core Bionic Composites: Preparation and Thermal, Compression, and Dynamic Cushion Properties. Polymers.

[25] Zhai W., Yu J., Wu L., Ma W., He J. (2006). Heterogeneous nucleation uniformizing cell size distribution in microcellular nanocomposites foams. Polymer.

[26] Sachse S., Poruri M., Silva F., Michalowski S., Pielichowski K., Njuguna J. (2014). Effect of nanofillers on low energy impact performance of sandwich structures with nanoreinforced polyurethane foam cores. J. Sandw. Struct. Mater..

[27] Metın D., Tihminlioğlu F., Balköse D., Ülkü S. (2004). The effect of interfacial interactions on the mechanical properties of polypropylene/natural zeolite composites. Compos. Part A Appl. Sci. Manuf..

